# Physical simulation study on grouting water plugging of flexible isolation layer in coal seam mining

**DOI:** 10.1038/s41598-022-04813-y

**Published:** 2022-01-18

**Authors:** Ang Li, Bingnan Ji, Qiang Ma, Yadong Ji, Qian Mu, Wenzhong Zhang, Pengfei Mu, Liang Li, Chunhu Zhao

**Affiliations:** 1 School of Architecture and Civil Engineering, Xi’an University of Science and Technology, Xi’an, 710054 Shaanxi China; 2 Coal Technology & Engineering Group Corp, Xi’an Research Institute of China, Xi’an, 710077 Shaanxi China; 3 Coal Technology & Engineering Group, Chongqing Institute of China, Chongqing, 400039 China; 4 Shaanxi Xiaobaodang Mining Co., Ltd., Shaanxi, 719000 Yulin China

**Keywords:** Sustainability, Civil engineering, Freshwater ecology

## Abstract

Deep coal seam mining often leads to water resource loss due to bedrock water entering the workings of the mine and is discharged adjacent to the mining area. Using the geological conditions of the Maiduoshan coal mine, this paper applied a physical simulation experiment. The specified rock above the coal seam was hydraulically fractured in advance to form a postmining grouted fracture network, followed by grouting to construct a flexible isolation layer that blocked the infiltration of groundwater from the aquifer into the water-conducting fracture zone. Stress sensors, flow sensors and strata displacement monitoring technology were deployed inside the experimental material to study the spatial distribution characteristics and evolution law of the water-conducting fracture zone in the overlying rocks. Analysis of the water-conducting fracture zone development law, stress variation, overburden evolution characteristics, fracturing and grouting sequence of the flexible isolation layer and the effect of postmining grouting on the water barrier was conducted. These experiments verified the feasibility of fracture and grouting of the flexible isolation layer. These research results will provide practical guidance for the transition from the current safe and efficient mining methods to safe and green mining methods of deep coal mining in the western mining areas of China.

## Introduction

With the large-scale and efficient mining of shallow resources, some mines have entered the deep mining stage^1,2^. Based on a preliminary statistical analysis of the hydrogeology and mining methods of large coal bases in Shendong, Shanbei, and Ningdong in western China, the overburden structure and ground stresses of the deep coal resources mined are significantly different from those of shallow mining. The flow field of direct and indirect aquifers, conditions of recharge, flow and draining, and evolution of water quality in deep mining all have complicated variations. In particular, effects on the ecological water table and surface ecology of the system can occur^3,4^). Most of the mining areas in western China have water shortages and fragile ecological environments^5–8^. Deep mining disrupts the overburden structure and causes bedrock water to enter the workings of the mine along with water-conducting fractures, resulting in discharge and loss of water resources. Furthermore, these processes destroy the groundwater circulation system affecting the surface ecology^9,10^.

In China, technical research regarding the mining and safety of shallow coal resources has preceded surface ecological protection, resulting in a large amount of water resource loss and ecological damage^11,12^. Therefore, deep coal mining in western mining regions require the protection of water resources and surface ecology, while ensuring safe and efficient mining operations^13^. Deformation and fracture of deep mining overburden and rock movement patterns are required from the perspective of mine production and safety,on the other hand, from the perspective of groundwater flow fields, it is necessary to master the characteristics of the groundwater flow field and evolution of mining water-filled aquifers.

Many scholars have conducted studies on water resource loss as a result of deep coal mining^14–16^. Gu^17,18^ proposed the establishment of a groundwater reservoir storage system, which can store groundwater in the voids of rock formations at the goaf by constructing a dam. Wang et al.^19,20^ proposed a conditional partition method to protect water during mining by developing mining methods for various coal seam characteristics to control groundwater level decline. Based on the water protection mining concept, Fan et al.^21^ proposed a high-intensity coal mining methods for the roof and floor, effectively protecting water resources in shallow aquifers. Stachowski et al.^22^ proposed concepts for water reclamation and the development of postmining areas after the Adamów Lignite coal mining was terminated, which can effectively increase the local water resources. Ma et al.^23^ evaluated the hydraulic connection between the Gaozhuang coal mine and the overlying lake and successfully applied overlying sandstone burst water control techniques to these coal seams. Although the abovementioned scholars have employed several methods to preserve water for coal mining, the concept of constructing a flexible isolation layer to prevent the loss of shallow water resources has not been proposed. By constructing a flexible isolation layer, the mining failure zone can be transformed, the influence range of mining fissures can be controlled, and the water environment conditions that satisfy the natural circulation of groundwater and surface ecology will be maintained or rebuilt. Using this method, the groundwater and surface ecology after mining can be maintained or even improved over the original state. This method is direct and convenient and can provide a new and effective method for the protection of shallow water resources in coal seam mining.

There are various methods used to study coal seam roof failure and the levels in aquifers due to the development of water conduction fracture zones. However, considering the difficulty of implementing field observations and the complexity of theoretical calculations to reproduce the deformation process of rock seams, including the fracture and grouting process of flexible isolated layers, it is best to use physically similar simulations for analysis. With the advantages of having a simple operation, clear and intuitive observation results, and short cycle time, physical simulations are now an essential research method in studying rock movement control science^24–27^.

Accordingly, this thesis therefore uses the deep coal seam mining conditions of the Maiduoshan coal mine and simulates the key technology of "flexible isolation layer" isolation reconstruction through physical similarity simulation experimental methods. Analysed the synergistic relationship between the mining stress field in the bedrock layer, overburden fracture field and mining disturbance seepage field. The simulation reproduces the spatial distribution characteristics of the water-conducting fracture zone, and the evolution process of the "flexible isolation layer" below the aquifer is simulated during mining. This research provides guidance for the protection of water resources in western mining areas of China.

## Engineering hydrogeological conditions

The Maiduoshan coal mine is a major mines in the Ningdong coal base in the western mining area that has entered the deep mining phase; located in the north–south reversible tectonic zone of the North China Platform and the folded and alluvial zone of the Ordos Basin. The fracture and fold tectonics are well developed and large in scale. With a total thickness of 358.25 m, the coal stratum in the Maiduoshan minefield is the Jurassic Middle Yanan Formation. The main lithologies are grey–white medium and coarse sandstone and grey and dark grey siltstone. The No. 2 coal located in the upper part of the Yanan Formation subcycle serves as the most dominant mineable coal seam in the minefield.

**Figure 1:**
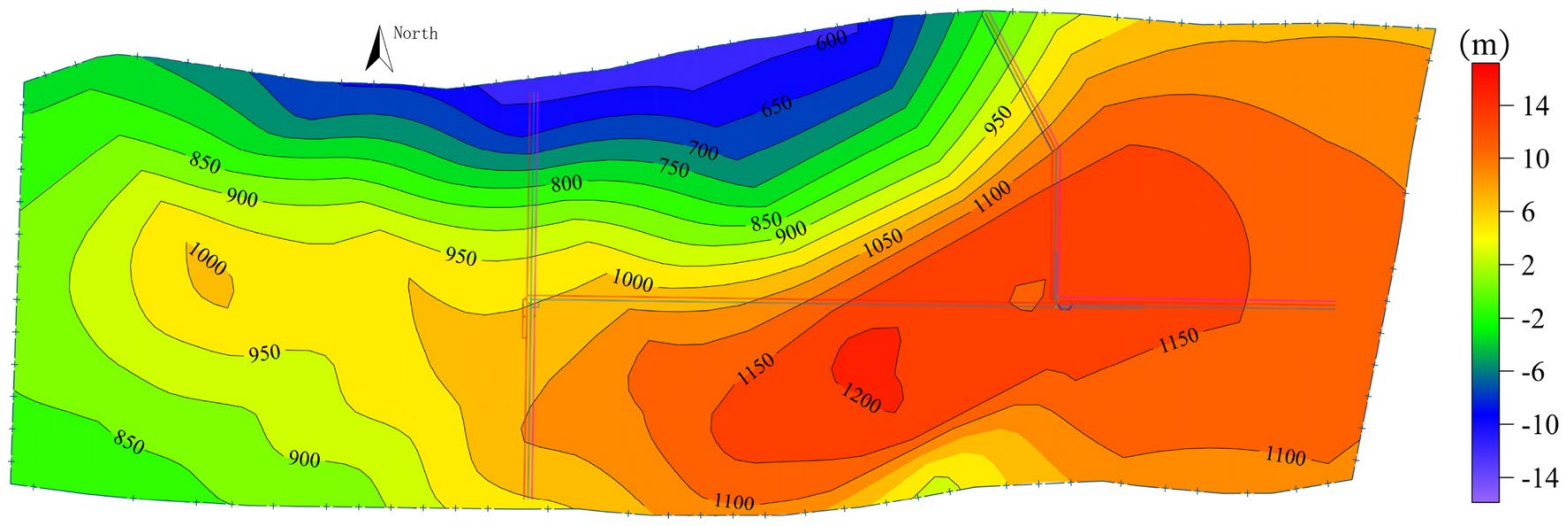
Water level contours in the No. 2 coal roof aquifer.

The fissure pore aquifer of the Naoro Formation in the Middle Jurassic is the main aquifer that influences mining at coal seam No. 2; the water level elevation is shown in Fig. 1. The sandstone at the bottom of this layer is relatively stable and mainly consists of coarse-grained sandstone, which is the direct top plate of the No. 2 coal seam. According to the sedimentary rotation, lithological characteristics and hydrogeological features of the formation, the gritstone at the bottom of the Naoro Formation is the main symbol layer, which divides the aquifer into upper and lower sections. Within the mining field, the roof water-resisting layer of the sandstone aquifer at the bottom of the Naoro Formation generally exists, blocking the hydraulic connection between the upper and lower sandstone aquifers of the Naoro Formation. This behaviour enables the lower sandstone aquifer of the Naoro Group sandstone to be a direct water-filled aquifer for the main mining 2 coal seam. On the other hand, the upper sandstone aquifer of the Naoro Formation is an indirect water-bearing aquifer and has little influence on the mining of the coal seam. The presence of siltstone and mudstone with water-resistant properties at different depths makes the vertical hydraulic connection of the aquifer extremely weak. Groundwater hydraulic flow fields are predominantly controlled by laminar flow.

Hydraulic conductivity between the aquifers occurs once the height of the two zones, formed because of rock movement caused by mining, reaches the erosion surface or the overlying water-rich layer. The fault fracture zones of the eastern and western boundaries have poor stratigraphic stability, high lithological variability and large differences in water-bearing properties. Therefore, the mining of 2 coal samples will inevitably lead to changes in the initial groundwater flow field in the mine area, resulting in a hydraulic connection between the upper and lower sections of the Naoro Formation sandstone aquifer. This in turn leads to changes in hydrogeological conditions in the lower section of the aquifer. Through the calculation of the height of the No. 2 coal caving zone and fracture zone, it reached the sandstone aquifer group at the bottom of the Naoro Formation. Groundwater will then enter the coal strata along the fracture zone, resulting in the loss of shallow water resources. A survey found that the water surge amount in Ningdong's coal mines was greater than 50 m3/h, and the pit fill source was dominated by roof aquifers and the accumulation of water ponded in the mining area. Therefore, according to the hydrogeology of Maiduoshan, the position of the flexible isolation layer is selected in the coarse-grained sandstone layer at the bottom of the Zhiluo Formation.

## Materials and methods

### Novel simulation materials

In response to the shortcomings of conventional similar materials, such as long drying time and nonreproducibility, a new block of similar material with a concave groove structure on three sides was developed. The block use the material as the module and cementing material as the intermodule binder. The production process of block material: mixing by cement, sand and latex or polymer in different proportions. The mixture was put into a supporting mould with built-in steel bars, and the top of the mixture after loading was smoothed. After a certain period of maintenance, the mould was removed, and the block was put into the maintenance box for 28 days. Because the blocks are not bonded, in practice, a separate binder has to be prepared and filled in the grooves between the blocks. The binder is made of different proportions of white powder, gypsum, sand and admixtures mixed with water to simulate the mechanical similarity between the blocks.

**Figure 2:**
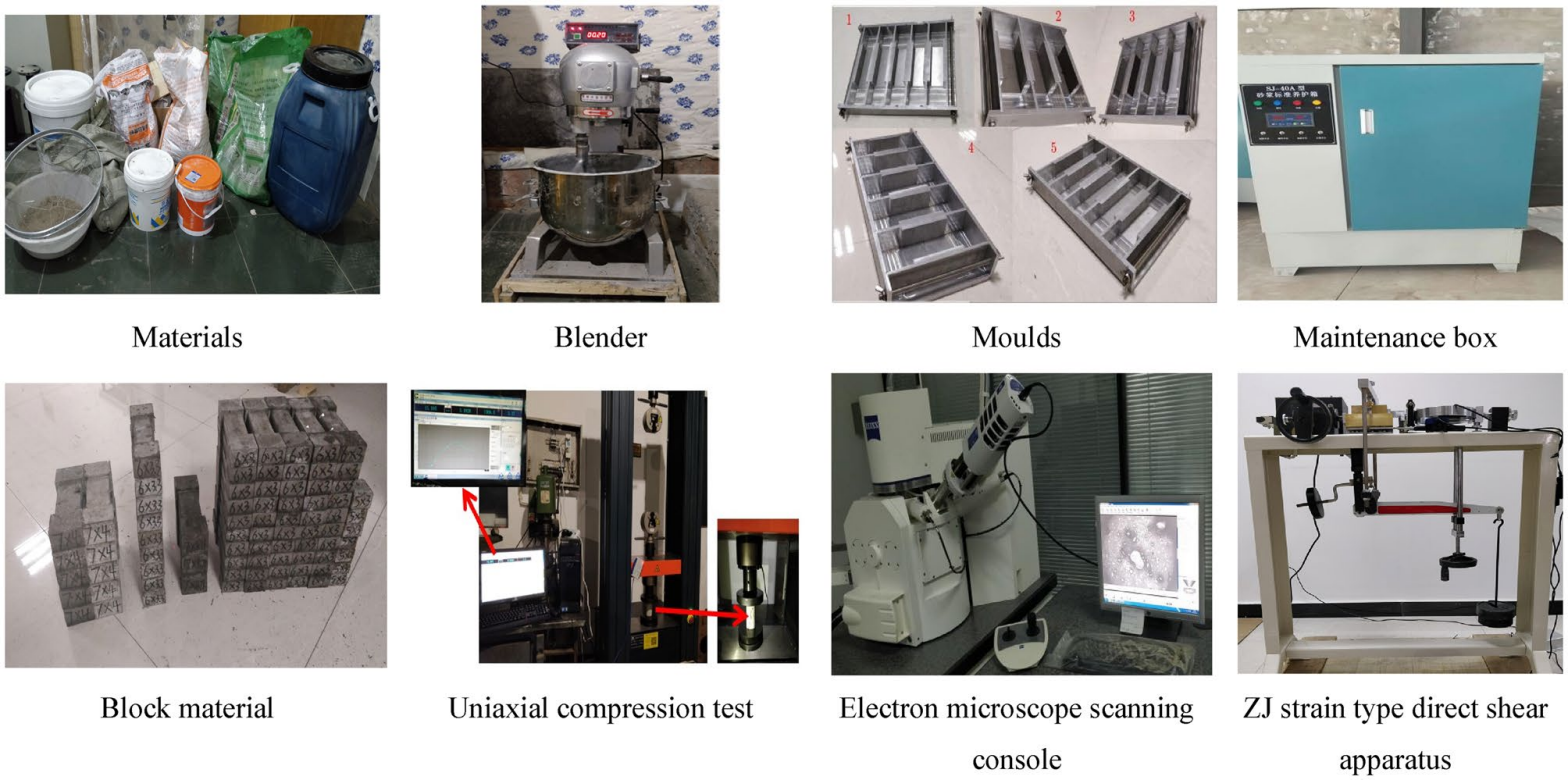
Production, maintenance and testing methods of materials.

Uniaxial compression, direct shear and electron microscopy scanning experimental results of the two materials in different ratios showed that when latex was added to the block material, cement reacted with the two-component latex waterproof material, which had high strength and was compactable. Adjusting the proportion of sand, the elastic model of the material changes accordingly and can be used as a reference to simulate sandstone. When the polymer was added to the block material, the elongation of the material increased as flexible chains of the polymer resisted external stresses. The material could also be selected to simulate mudstone strata. By varying the ratio, the cohesive strength and internal friction angle of the cementing material also showed the appropriate changes to simulate different lithologies, such as soft to hard rock layers in the strata. Mica powder was used to simulate the stratification between the various rock layers. In similar to true geological conditions, the mechanical property parameters, such as the elastic modulus of similar materials and the cohesion, internal friction angle and flexural strength of cementing materials, satisfy a similar theory. Based on the mechanical property parameters of the coal seam and overburden roof in the model, similar materials of different formulations and ratios were selected in conjunction with the mechanical property tests of similar material samples, including the material production, maintenance and testing methods are shown in Fig. 2. The mechanical parameters of similar materials are listed in Table 1.

**Table 1:** Simulation parameters for native rock and similar materials.

Rock lithology	Native rock			Similar material						
	Elastic modulus (GPa)	Cohesion (MPa)	Strata thickness (m)	Cohesion (MPa)	Elastic modulus (MPa)	Strata thickness (mm)	Block material elastic modulus (GPa)	Cementing materials cohesion (MPa)	Block material ratio	Cementing materials ratio (Sand:gypsum:white powder:water)
**Naoro Formation lower section**										
Gritstone	45.7	3.96	12.7	24.76	285.7	95.6	284.3	25.99	Cement:latex:sand = 1:0.1:4	4:2:1.5:2
Siltstone	44.4	1.94	10.6	12.14	277.4	79.6	278.1	12.88	Cement: JS polymer compound = 1.2:1	1:2:1:1
Gritstone	45.7	2.82	10.1	17.65	285.7	76.6	284.3	17.27	Cement: latex:sand = 1:0.1:4	2:2:1.5:1.5
**Yan'an Formation**										
Medium grained sandstone	59.5	3.42	2.2	21.37	371.7	16.6	365.3	20.80	Cement:latex:sand = 1:0.5:2	2:2.5:1.5:1.5
Siltstone	40.0	4.30	1.7	26.85	250.0	12.6	254.2	24.60	Cement:latex:sand = 1:0.2:3	4:2:1:1.5
Mudstone	10.5	0.41	0.8	2.56	65.5	6	54.6	3.84	Cement: JS polymer compound = 1.0:1	3:2:1:1
Siltstone	42.2	2.40	0.4	15.00	263.8	3	254.2	14.50	Cement:latex:sand = 1:0.2:3	2:1.5:1.5:1.5
No.2 Coal seam	3.3	0.84	0.2	5.24	20.8	1.5	17.9	6.90	Nail free adhesive	1:1:1:1

### Material of flexible isolation layer

**Figure 3:**
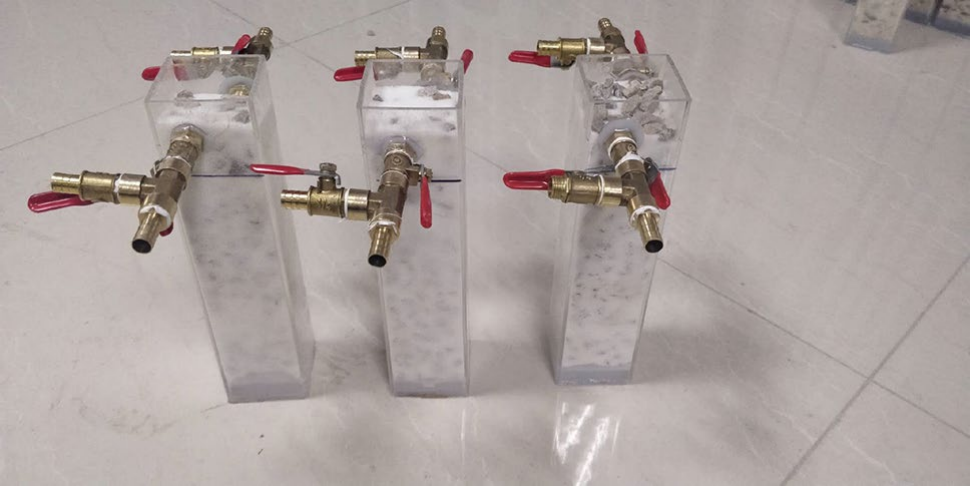
Flexible isolation layer.

**Figure 4:**
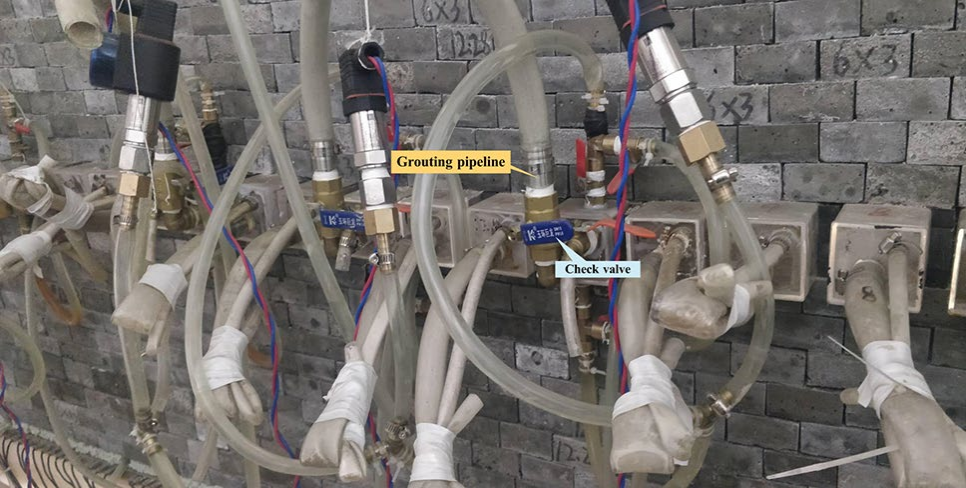
Isolation layer grouting reformation system.

A specific aquifer in the upper part of the coal seam is used as a flexible isolation layer, and the layer is hydraulically fractured before mining to form a specific thickness of the artificial fracture development zone. After full coal seam mining, the isolation layer is reformed by grouting to control the impact range of mining fractures and interrupt the downwards seepage of the aquifer. Therefore, the selection of the isolation layer material is particularly important in physical similarity simulation experiments to meet the conditions of the similarity theory and to achieve the conditions for fracturing, seepage and grouting. After a series of material selections and experiments, a high-strength rectangular transparent plastic was chosen as a similar material for the isolation layer, as shown in Fig. 3. The elastic modulus of the material is 371.7 MPa, and the size is 50 mm × 250 mm × 50 mm (length × height × depth). The unit was filled with 5–10 mm gravel and iodized salt in a 3:1 ratio, with single inlet and double outlet valves on the top and bottom of the unit. After the completion of the model, the unit body was washed with hot water before mining by using the characteristics of iodized saltwater regularization. The cuboid is completely dissolved and the iodized salt released can simulate the isolation layer after fracturing. After the isolation layer is fractured, the isolation layer water flows down the monitoring line, simulating the aquifer water flowing down the rock fracture. The grouting reformation system of the isolation layer comprises a large diameter unidirectional valve at the end of a similar material in the isolation layer, grouting pipeline, slurry and manual grouting machine, as shown in Fig. 4. The inorganic waterproof plugging material is selected as the grouting material, which is mixed according to the water cement ratio of 0.3:1 at room temperature, followed by using a manual grouting machine to construct the isolation layer. The slurry can be completed within 3 min of the initial set and 6 min after the final set. After the slurry is solidified, the water cannot flow along the monitoring line to simulate the sealing of the fissure after the isolation layer grouts on site, thus achieving the water plugging effect of grouting from the isolation layer.

## Platform construction

### Similarity ratio

The similarity simulation experiment adopts the material with the strength to simulate the actual rock stratum. The similarity between the simulated rock and actual rock stratum should be guaranteed in terms of mechanical strength.

#### Geometric similarity ratio

Geometric similarity is a certain proportional relationship between the simulation model and the actual rock mass prototype in space. Thus we use the geometric similarity ratio *C*_*L*_:

1 $$ C_{L} = \frac{{L_{H} }}{{L_{m} }} = \frac{{L_{H} }}{{L_{m} }} = 140 $$

Among them: *C*_*L*_—geometric similarity ratio; *L*_*H*_- Geometric size of prototype along the actual strike length of rock stratum, cm, cm; *L*_*m*_—The geometric size of the model along the direction of the simulated rock strike length, cm; *L*_*H*_—Geometric size of prototype along the actual cumulative thickness direction of rock stratum, cm; *L*_*m*_—Geometric size of the model along the vertical height direction of the simulated rock stratum, cm.

#### Bulk density similarity ratio

2 $$ C_{\gamma } = \frac{{\gamma_{H} }}{{\gamma_{m} }} = 1.2 $$

Among them: *C*_*γ*_—bulk density similarity ratio; *γ*_*H*_—apparent density of original rock, g/cm^3^, *γ*_*m*_—Apparent density of similar material, g/cm^3^.

Similarity ratio of stress, strength, elastic modulus, head pressure and cohesion (*C*_*R*_)

According to the basic principle of similarity theory, the similarity ratio of stress, strength, elastic modulus, head pressure and cohesion is as follows:

3 $$ {\text{C}}_{{\text{R}}} = \frac{{{\text{R}}_{{\text{H}}} }}{{{\text{R}}_{{\text{m}}} }} = \frac{{{\text{E}}_{{\text{H}}} }}{{{\text{E}}_{{\text{m}}} }} = \frac{{{\text{P}}_{{\text{H}}} }}{{{\text{P}}_{{\text{m}}} }} = \frac{{{\text{C}}_{{\text{H}}} }}{{{\text{C}}_{{\text{m}}} }} = \frac{{\gamma_{{\text{H}}} }}{{\gamma_{{\text{m}}} }} = {\text{C}}_{{\text{L}}} \times {\text{C}}_{\gamma } \times {\text{C}}_{{\text{L}}} = 168 $$

Among them: *R*_*H*_, *E*_*H*_, *P*_*H*_, *C*_*H*_—uniaxial compressive strength, elastic modulus, head pressure and the cohesion of original rock, MPa; *R*_*m*_, *E*_*m*_, *P*_*m*_, *C*_*m*_ -Uniaxial compressive strength, elastic modulus, head pressure, cohesion of similar materials, MPa.

#### Similarity ratio of strain, Poisson ratio internal friction angle $\left( {C_{\varepsilon } = \frac{{\varepsilon_{H} }}{{\varepsilon_{m} }} = \frac{{\mu_{H} }}{{\mu_{m} }} = \frac{{\phi_{H} }}{{\phi_{m} }} = 1} \right)$

4 $$ \left( {C_{\varepsilon } = \frac{{\varepsilon_{H} }}{{\varepsilon_{m} }} = \frac{{\mu_{H} }}{{\mu_{m} }} = \frac{{\phi_{H} }}{{\phi_{m} }} = 1} \right) $$

Among them: $\varepsilon_{H} ,\;\mu_{H} ,\;\phi_{H}$ -Strain, Poisson ratio and internal friction angle of the original rock; $\varepsilon_{m} ,\;\mu_{m} ,\;\phi_{m}$-Strain, Poisson 's ratio and internal friction angle of similar materials.

#### Time similarity ratio (_*CT*_)

In this similar simulation test, the stress, displacement of the overlying rock roof and the evolution process of rock fracture and collapse in the process of coal seam mining change with the advancement of the working face. A similar simulation process should be similar to the actual mining conditions in the field and meet the similarity ratio in time.

5 $$ C_{T} = \frac{{T_{H} }}{{T_{m} }} = \sqrt {C_{L} } = 11.8 $$

Among them: *T*_*H*_—the time required for coal seam mining in actual situations, d; $\varepsilon_{m} ,\;\mu_{m} ,\;\phi_{m}$—time required for mining of the working face in the related model, d.

The daily advance of the Maiduoshan No. 2 coal seam is 16.8 m, which is equivalent to a 15 mm advance in 15 min according to the time similarity ratio, so each block can be made into 15 mm blocks.

### Similar simulation experimental platform construction

**Figure 5:**
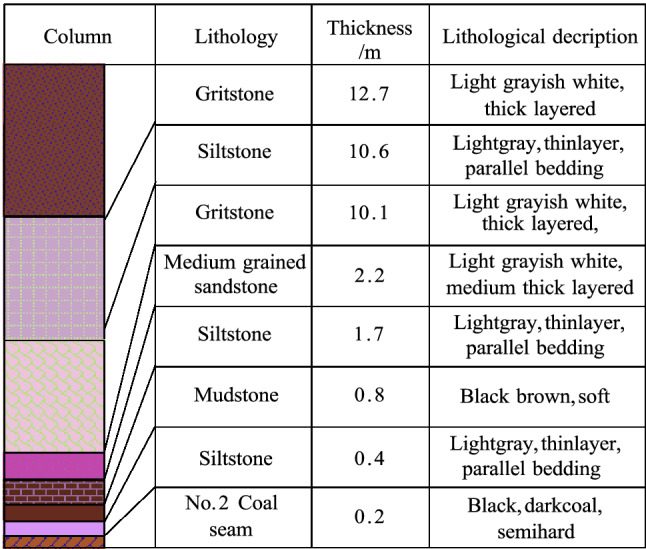
Drilling histogram.

Combined with the hydrogeological conditions of the Maidushan Coal Mine and the relevant requirements of the test, the No. 2502 drilling hole was selected with a surface elevation of 1401.39 m. The No. 2 seam has an average thickness of 3.2 m, a burial depth of 491.2 m and a strike length of 250 m. The altitude of the gritstone aquifer in the lower section of the Naoro Formation is 1304.11 m, with a distance of 403.92 m from the roof of the No. 2 coal seam and a bottom water pressure of 3.7 MPa. The model dimensions were conceived as 2500 (length) × 200 (width) × 1000 mm (height). Given the influence of the model boundary, 450 mm coal pillars were left on both sides of the model. The number of excavation steps was 157 by imposing the surface force of the model to replace the overburden weight of the roof. The flexible isolation layer was divided into 31 units. To create the model, the stress sensors were first placed in the lowest layer, and the layers were laid in sequence. Seven aqueducts (i.e., monitoring line) were placed in the rock lays from left to right to test the water flow. To achieve the independent application of static load, steel bars were laid on the top of the model to help simulate the weight of the uppermost overburden layer to solve the phenomenon of uneven roof pressure caused by mining subsidence in the conventional model. By varying the weight and number of steel bars, the weight of different roof overburdens can be achieved. The borehole histogram is shown in Fig. 5.

### Water pressure control system

**Figure 6:**
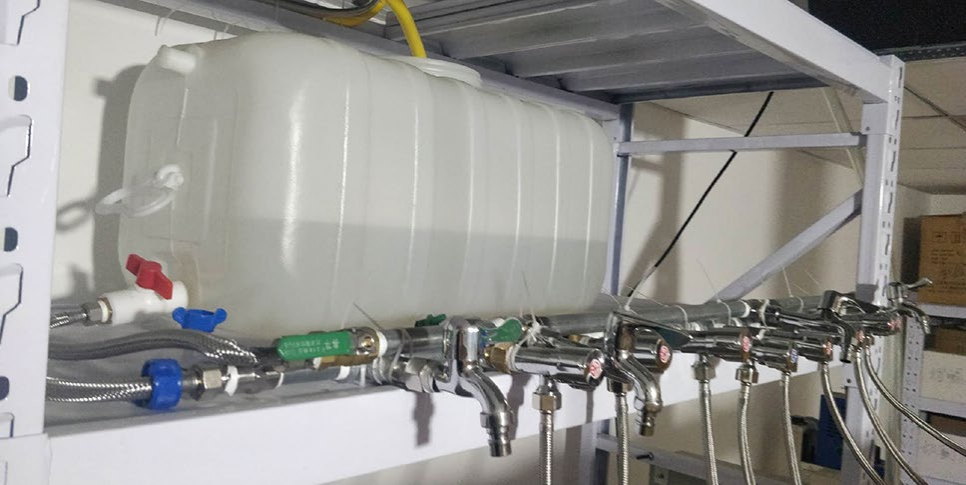
Water pressure control system.

The water pressure control system consists of a water storage tank, a water temperature controller, a water level monitor and various shunt pipelines. In accordance with similar theoretical principles, the water storage tank is placed on a shelf at a certain height to simulate the water pressure of the aquifer, as shown in Fig. 6. The height of the storage tank on the shelf can be modified to provide different amounts of water pressure. The external water source is heated to 65° by the water temperature controller and then injected into the water tank. The water level monitor probe is positioned inside the water tank to monitor the water level inside the water tank in real time, thus ensuring stable water pressure. The distribution pipeline at the outlet of the water tank is attached to the single inlet and double outlet valve heads on the isolation layer through the fine rubber tube and then buried in the model with water pressure sensors and flow metres attached to the outside of the pipeline. During the mining process, the water flow inside the model is observed using an infrared thermal imager, and together with the detection data of sensors and flowmeters, the crack development and stress release degree of roof strata can be analysed.

### Date collection

**Figure 7:**
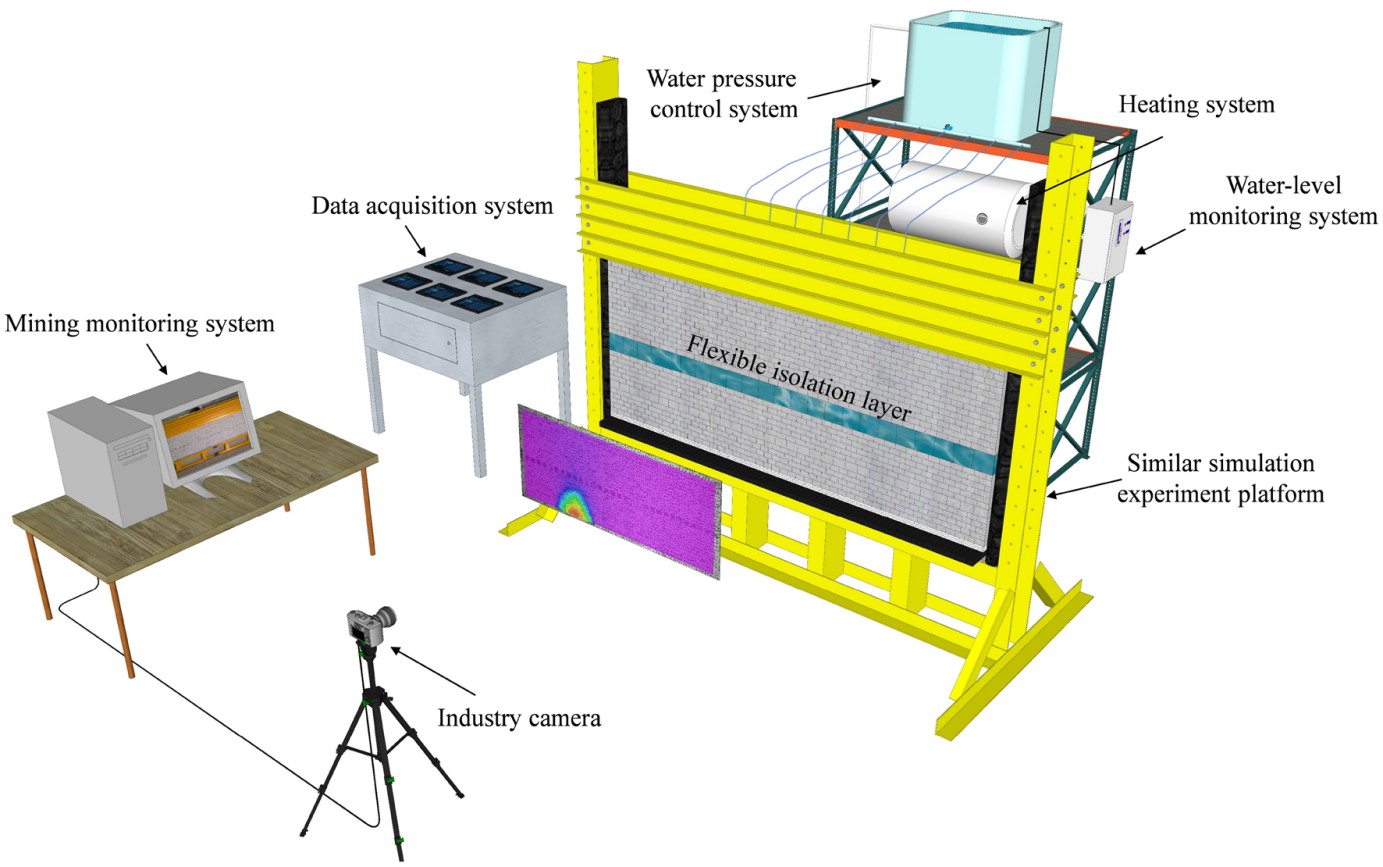
Experimental platform assembly diagram.

**Figure 8:**
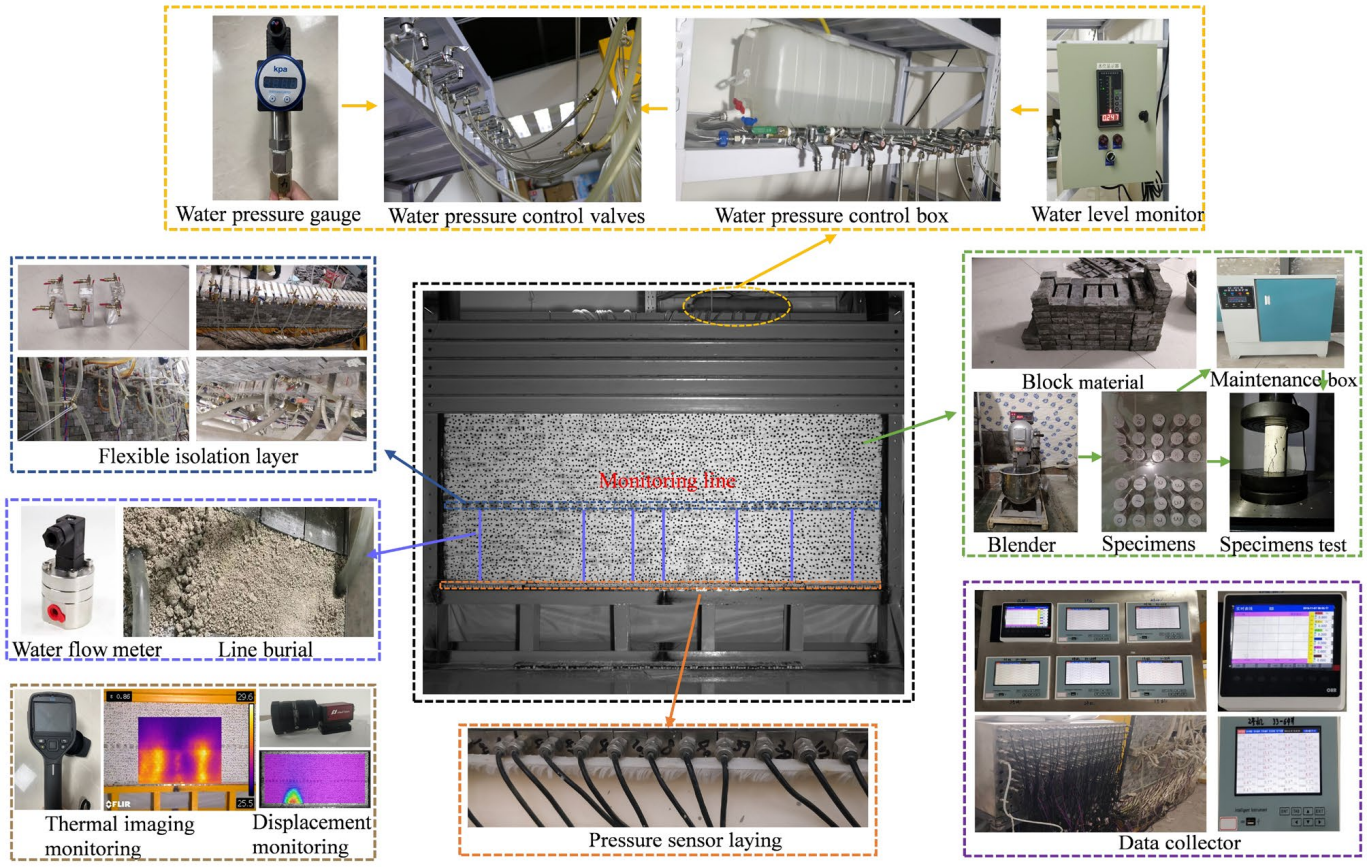
Physical diagram of the experimental platform.

The experimental simulation used a high-precision scattering technique to monitor the physical simulation surface displacement with supporting full-field strain measurement and simulation optimization analysis software to conduct real-time diagnosis of the displacement changes in the roof rock seam. The data acquisition system consists of stress sensors, water flow metres and collection terminals. There are 118 stress sensors arranged below the coal seam to test the real-time change pattern of the roof rock surrounding stress and infer the periodic incoming pressure on the roof. According to the water flow monitoring in the seepage line by the collection terminal, the fracture development of the roof water-conducting fracture zone can be analysed. After the completion of the grouting transformation, the change in flow rate can also be evaluated by the grouting effect. Details of the experimental platform are shown in Fig. 7 and Fig. 8.

## Experimental procedures

1. The adjusted stress sensor was laid at the bottom of the test bench, and the guard plates were clamped on both sides. The coal seam was laid above the sensor, and then the corresponding prefabricated block simulation material was placed above the coal seam in turn. Sprinkling mica powder between different lithology rock layers was used to simulate the layered joints between rock layers. During the process of model laying, seven pipelines used to simulate seepage were buried in the model until the isolation layer was laid out.
2. After the completion of the model laying, the aquifer mining system, the isolated layer grouting reformation system, and the data collection system were connected. The baffle was then removed, and a layer of white powder was brushed onto the outside of the model. After that, the black spots were dotted on. The mining monitoring system was adjusted to check whether it could meet the requirements of real-time analysis.
3. After all the adjustments were completed, the first section of the isolated layer was first fractured, i.e., the hot water of the water tank was introduced into the flexible compartment, and then the coal seam was mined. When the roof strata were completely stable after each mining step, the model was photographed using an industrial camera and an infrared thermal camera and analysed by combining the collected terminal data and corresponding software to determine the changes in the displacement, stress and seepage field of the overlying strata.
4. When the impact range of roof deformation approaches the fracturing boundary of the first isolation layer (i.e., the corner of the bedrock impact boundary extends to the fracture range of the flexible isolation layer), mining is stopped, and the second section isolation layer is fractured. When the roof strata within the scope of the first isolation layer is reach full exploitation, the first isolation layer should be transformed by grouting the first isolation layer, mining should continue, and the above steps should be repeated until mining reaches the stop line.

## Results and discussions

### Analysis of the roof failure characteristics of coal seam

**Figure 9:**
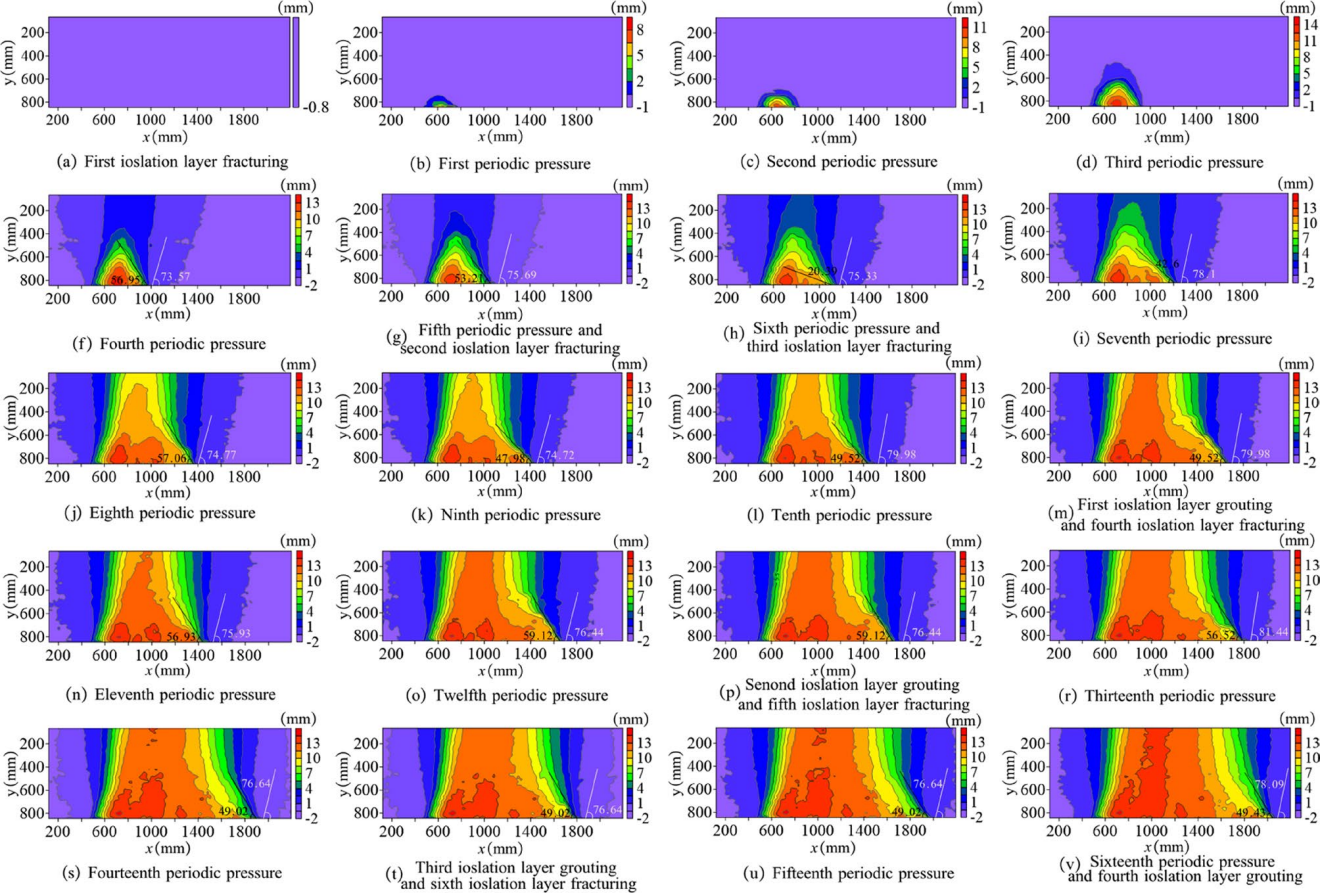
Influence law of coal roof rupture during different periodic pressure.

Before mining, fracturing was conducted on a portion of gritstone in the lower section of the Naoro Formation and then entered the mining stage. Figure 9 shows the influence law of coal roof rupture under different periodic pressures. With mining of the #2 coal seam working face, the direct roof of the coal seam partially broke and collapsed, forming gangue in the goaf. There is a clear separation between the direct and basic roof. When the working face advanced to 228.2 mm, the old roof ruptured, and the working face started to enter the periodic pressure-bearing stage. As the working face advanced to 592.9 mm, the roof exhibited the fourth periodic pressure. The overlying layer roof in the excavation area was affected by the upper bearing arch pressure, leading to the collapsed rock to not completely contact the upper roof. With the increasing distance of coal seam mining, the roof developed significant subsidence, and the influence range of the bedrock boundary caused by the mining was still in the isolation layer fracturing zone. The bedrock influence boundary angle reached 73.57°, and the rock fracture angle was 56.95°. When the working face advanced to 726.5 mm, the fifth periodic pressure on the roof occurred. The bedrock layer in the upper right of the workings was near the right boundary of the first isolated coal seam rupture. Then, coal mining was suspended, and a second isolated seam fracturing process was conducted. The bedrock influence boundary angle reached 73.57°, and the rock rupture angle was 56.95°.

When the processing was advanced to 798.4 mm, the bedrock layer in the upper right of the processed area became close to the right boundary of the second isolated seam fracturing. After the third isolated layer fracturing process, the rock impact boundary angle reached 75.33°, and the rock fracture angle was 50.39°. Proceeding to 1031.6 mm, eighth periodic pressure was generated on the roof. The falling gangue in the mined-out area was in contact with the roof, with the bedrock impact boundary angle reaching 74.77° and the rock fracture angle reaching 57.06°. Thereafter, the bedrock layer of the roof gradually entered the full-scale mining stage. As the working face continues to advance, the bedrock impact boundary caused by coal seam mining should be in isolated coal seam fractures. When the bedrock layer at the working face is close to the right boundary of the isolation layer fracturing, the next isolation layer fracturing should be performed.

### Analysis of roof stress evolution law

**Figure 10:**
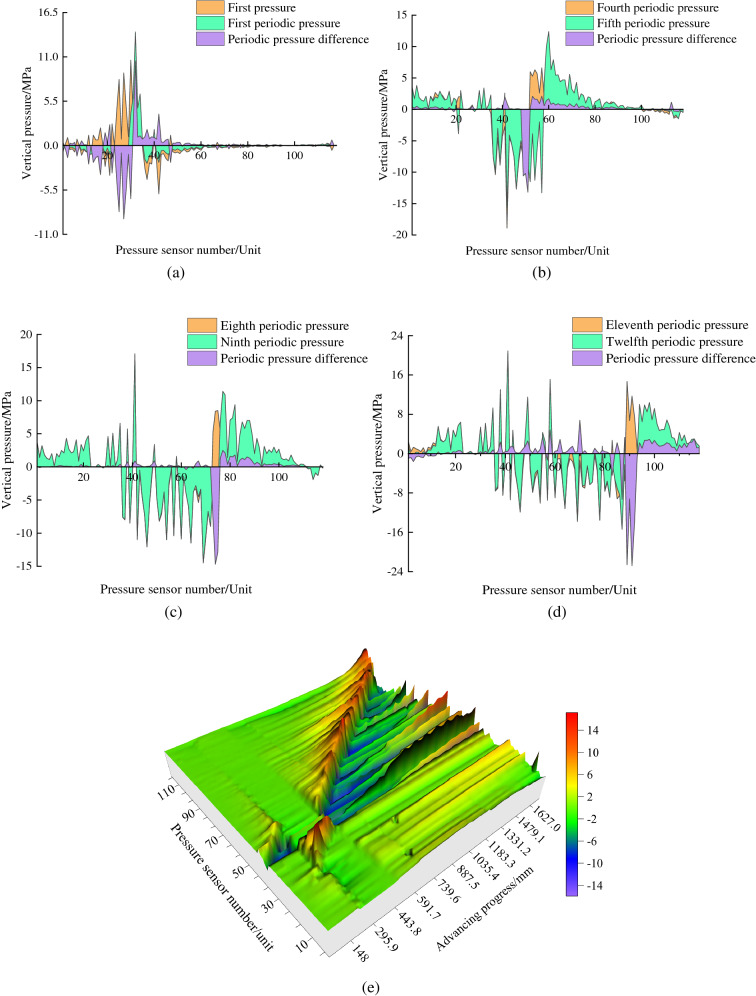
Vertical pressure variation law with coal mining. (**a**) First pressure and First periodic pressure difference. (**b**) Fourth and First periodic pressure difference. (**c**) Eighth and Ninth periodic pressure difference. (**d**) Eleventh and Twelfth periodic pressure difference. (**e**) Variation laws of vertical pressure with mining.

Figure 10 illustrates the change law of the roof support pressure when mining of the working face, in which the roof support pressure curve is the stress change minus the initial value of the sensor before mining. After the excavation of the working face, the surrounding rock will exhibit stress redistribution. The increase in tangential stress in front of the working face or on both sides is called the support pressure. The peak value of the support pressure generally occurs on the front of the working face. As the working face advanced to 228.2 mm, the direct roof gradually broke and collapsed with mining. Due to the redistribution of surrounding rock stress, the stress fluctuation at the open cut was clear. In front of the working face, the overlying rock stress was redistributed due to mining, and the vertical pressure peak area appeared, with a stress increment of 0.03 MPa. When the working face advanced to 360.8 mm, the first cycle pressure on the roof occurred. The falling gangue in the mine-out area gradually approached its upper strata, and the peak support pressure increments reached 0.05 MPa. During the advancement of the working face to 592.9 mm, the direct roof continued to collapse. The gangue at the cuttings was gradually compacted with the roof, and the stresses gradually restored to stability. Coal seam mining led to the decompression of the floor, and the vertical stress maximum reduction at the working face was 0.045 MPa. The peak vertical pressure in front of the working face shifted to the right as mining progressed. When degradation reached 726.5 mm, the fifth periodic pressure on the roof occurred. Figure 10b shows that the fracture of the isolation layer had no apparent effect on the change in roof stress. Within 560 mm from the open excavation, the mine-out area gangue gradually compacted with the roof. Vertical pressure changes between the fourth and fifth periodic pressures are slight and practically nonsignificant.

When the mining reached 1031.6 mm, the directly caving gangue completely filled the goaf and was compacted with the roof. The upper roof of the caving rock was supported again, and the compaction range of the mining area extended to 821 mm. As the working face advanced to 1338.9 mm, the peak vertical pressure appeared at 1400 mm, with a maximum increment of 0.375 MPa. The compaction range of the mining area extends to 1200 mm. Then, the fractured isolation layer can be grouted. The subsequent working face advances until the end of mining, and the rock movement above the mine-out zone will exhibit a periodic "falling-filling-cutting-compaction" process. Fracture grouting of the flexible isolation layer has no significant effect on the vertical stress changes, and the stress unloading area and the peak vertical pressure will continue to change with mining. Nevertheless, consideration needs to be given to the adequacy of the gangue falling from the roof for isolation layer grouting.

### Roof displacement and development pattern of water-conducting fracture zone

**Figure 11:**
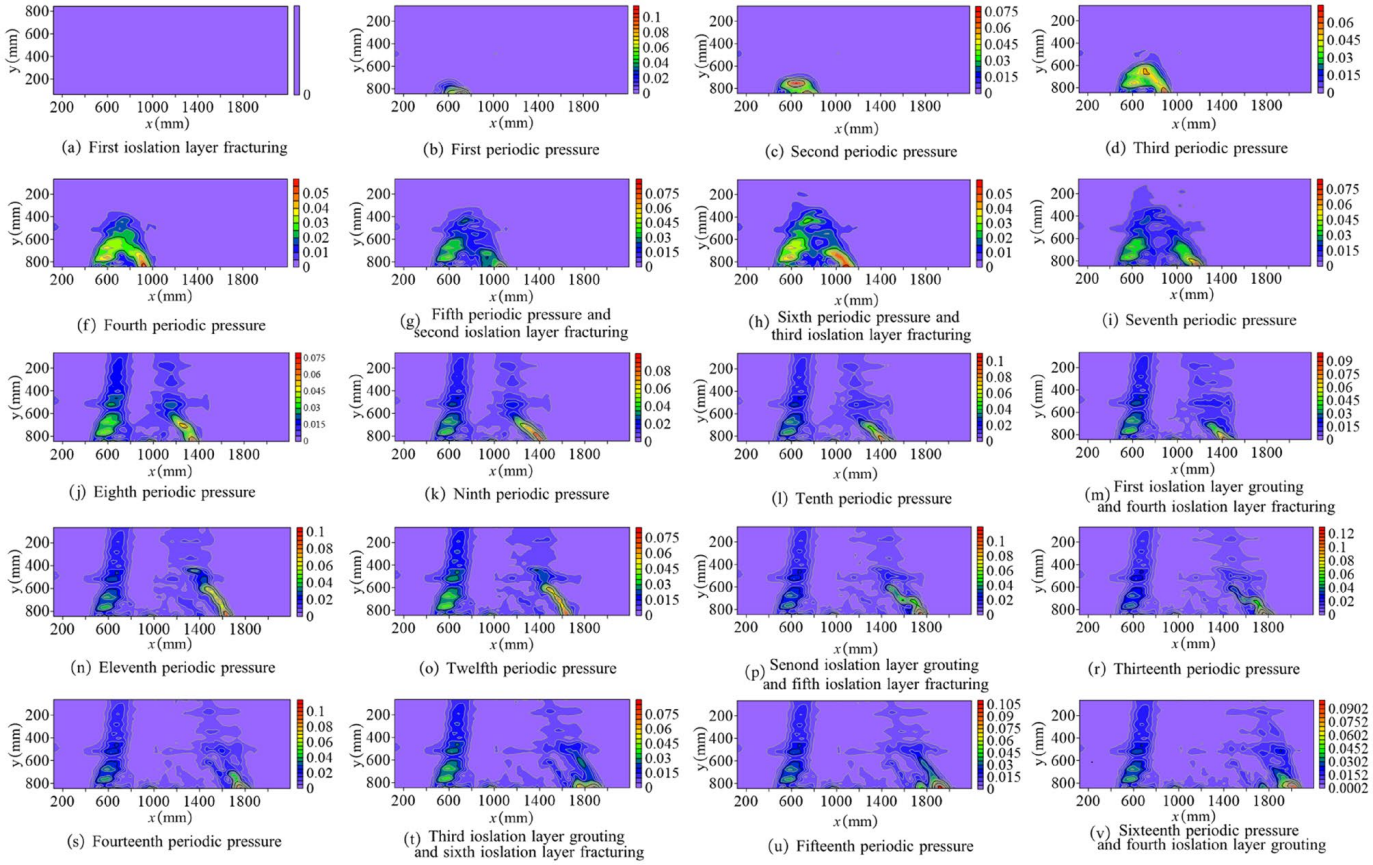
Development regularity of roof water-conducting fissures during different period pressure.

**Figure 12:**
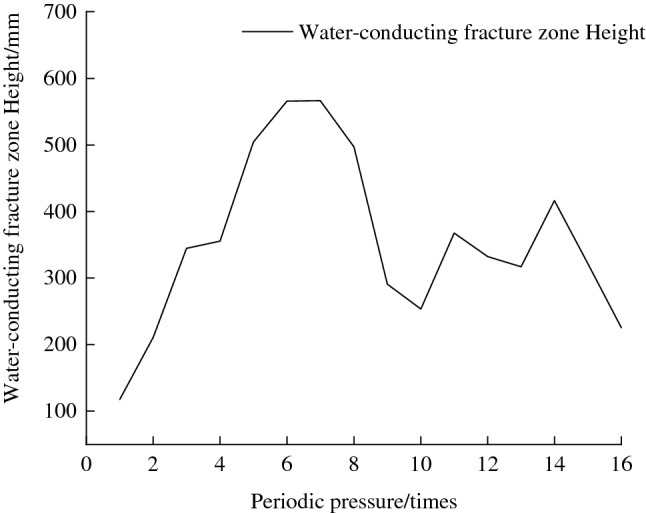
Development height curve of water-conducting fracture zone.

Figure 11 shows the development law of the roof water-conducting fissures in the roof of the coal seam during different pressure periods, where the illustration shows the von Mises equivalent strain. Figure 12 shows the development trend of the water-conducting fracture zone height. From the whole observation, although the isolation layer is treated by fracturing before back mining, it has less influence on the displacement and deformation of the overlying rock layer because it is restricted by the surrounding rock of the model. When the working face was mined to 228.2 mm, the upper roof of the mining face collapsed, and the first periodic pressure occurred on the roof. The roof displacement reached the Yan'an Group mudstone layer, and the roof collapse height was only 104.3 mm. As the mining advanced, the roof fractures in the mining-out area continued to develop upwards. When the working face was mined to 360.8 mm, the first cycle pressure on the roof occurred, and the roof collapse height extended upwards to the siltstone of the Yan'an Formation, with a collapse range of 117.6 mm. At this point, only a small displacement change occurred around the direct roof, and the flexible isolation layer was basically not affected by any impact.

From the second cycle pressure onwards, the development trend accelerated significantly, and the collapsed height rose rapidly to 210.9 mm. When the working face advanced to 537.1 mm, the third cycle pressure occurred on the roof. The collapsed Yan'an Formation mudstone layer was further pressurized by its upper layers and collapsed to a height of 344.7 mm. The roof displacement had spread to the coarse sandstone of the Naoro Formation, but the height of the water-conducting fracture zone had not reached the bottom of the isolation layer. When the workings reached 592.9 mm, the roof collapsed again, showing the fourth periodic pressure. The water-conducting fissure zone continues to develop upwards to 355.3 mm, which passes through the fissure isolation layer and reaches the gritstone at the top of the isolation layer. The fractured isolation layer is in an "activated" state.

When the working face reached 1031.6 mm, fallen gangue completely filled the mining-out area and compacted with the roof, and eighth periodic pressure occurred on the roof. The height of the water-conducting fracture zone developed to 496.8 mm, which was lower than the height of the water-conducting fracture zone of 565.8 mm at the seventh periodic pressure. After that, the old roof collapsed as a cantilevered beam. The development height of the water-conducting fracture zone was allegedly less than 565.8 mm. Afterwards, the roof fracturing direction was consistent with the direction of working face advancement, from left to right. Displacement and fracture of the overlying rock layer were mainly caused by the overall downwards sliding of the upper rock seam due to the collapse of the bottom rock seam. At different heights of the coal seam roof, the degree of displacement damage decreased with increasing height.

When the working face reached 1178.7 mm, the roof covering the open cut stabilized. The fractured isolation layers in the 1st ~ 13th groups were grouted, and then the coal was mined only after the slurry had completely solidified and reached a certain strength. The eleventh periodic pressure occurred on the roof, with a water-conducting fracture height of 367.6 mm at this time. When the working face was advanced to 1471.9 mm and 1645.2 mm, the roof had twelfth and fourteenth periodic pressures, and the heights of the water-conducting fracture zone were 332.0 mm and 416.0 mm, respectively. Then, the 14th ~ 15th and 16th ~ 17th group isolation layers of the upper coal seam were grouted while fracturing the right isolation layer. However, the disruption of displacement towards the extent of the development had a relatively small impact, mainly on the roof rock layer above the mining face. Table 2 indicates the development height of the water-conducting fracture zone and the fracture and grouting sequence of the isolated layer.

**Table 2:** Development pattern of water-conducting fracture zone and fracture and grouting sequence of isolated layer.

Mining step	Mining advancement (mm)	Periodic pressure	Isolated layer fracturing	Isolation layer grouting
0			Groups 1 to 19	
47	228.2	initial weighting		
56	360.8	1		
61	432.7	2		
68	537.1	3		
72	592.9	4		
81	726.5	5	Groups 20 to 21	
86	798.4	6	Groups 22 to 25	
92	886.7	7		
102	1031.6	8		
106	1090.4	9		
112	1178.7	10	Groups 26 to 27	Groups 1 to 13
123	1338.9	11		
132	1471.9	12	Groups 28 to 29	Groups 14 to 15
136	1528.6	13		
143	1645.2	14	Groups 29 to 31	Groups 16 to 17
147	1686.6	15		
153	1774.9	16		Groups 18 to 20

During the mining process, damage to the water-conducting fissure zone was always a major factor in the displacement of the roof slab. Nonetheless, after fracturing and grouting measures, the effects of the damage were significantly reduced such that the damage to the roof rock was contained within the flexible isolation layer. After grouting, the enhanced strength of the isolation layer ensured that mining was carried out normally. During the mining period, four grouting reforms were made, and the isolation layer was fractured six times, with the maximum development height of the water-conducting fracture zone located at the seventh periodic pressure, reaching 565.8 mm.

### Analysis of water flow evolution law of overburden roof

**Figure 13:**
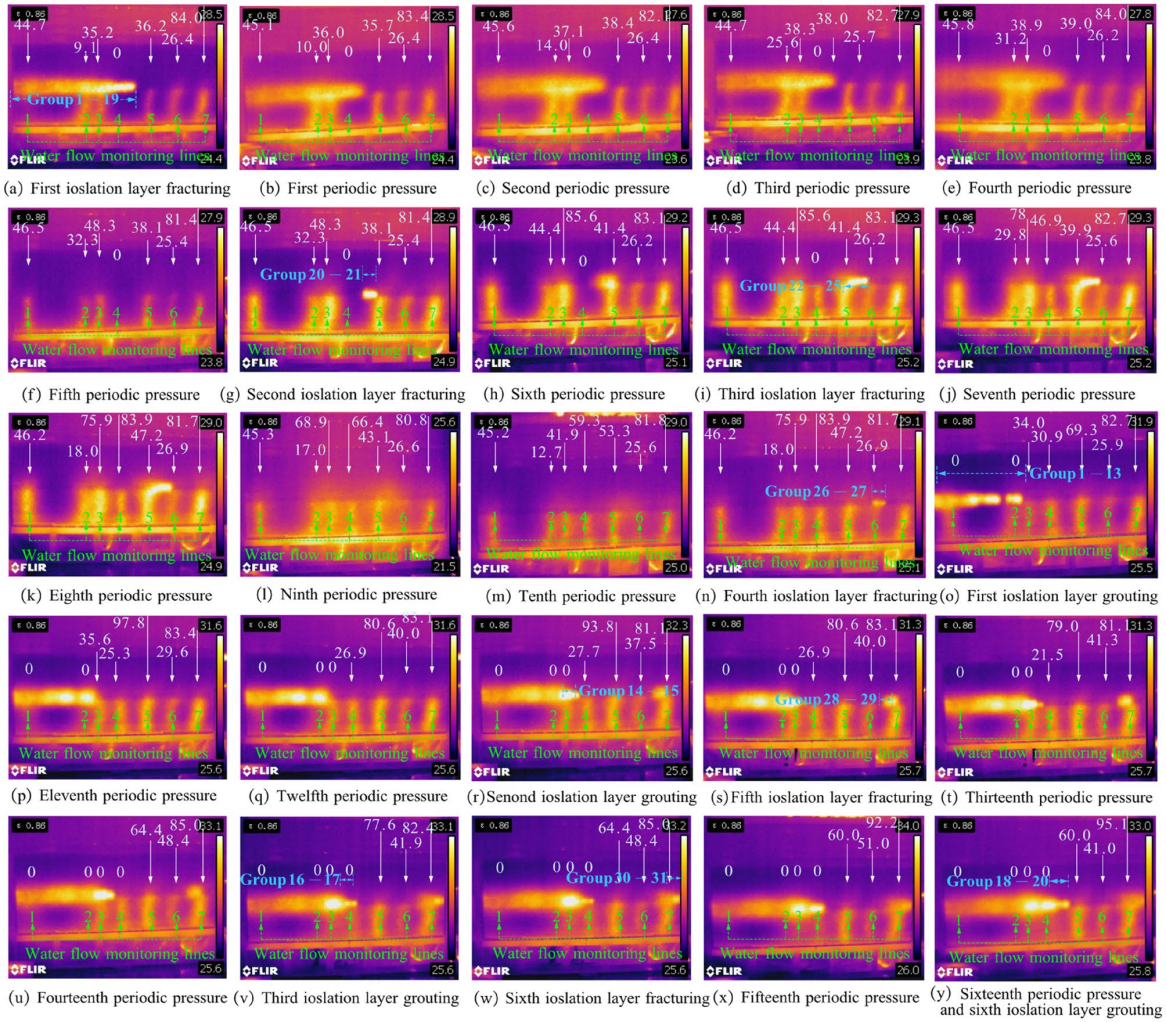
Water flow evolution of the overburden roof with coal mining.

To analyse the seepage law of the overburden roof, seven water flow monitoring lines were arranged from the top of the flexible isolation layer to the direct roof of the coal seam. The No. 1 water flow monitoring line was placed in the position of the third group of the isolation layer, which is initially located outside the deformation range of bedrock disturbed by mining and outside the stop line. The flow line was mainly used to monitor the influence of the rock disturbance boundary above the open cut on isolated seam fracturing and grouting. No 2–3 water flow monitoring lines were placed at the isolation layer positions of Group 12 and Group 14, which were initially located near the maximum height of the water-conducting fracture zone and were mainly used to monitor the change laws of the water-conducting fracture zone with mining impact. Monitoring Lines 4–6 were placed in isolation layers No. 17, No. 22 and No. 26 to study the impact of water flow changes with mining disturbance and the advanced influence scope. Water flow monitoring line No. 7 was placed in the thirtieth group of isolated layers, which was originally outside the cut-off line. As shown in Fig. 13, white arrows are water flow vectors in mL/min. Fracturing the 1–18 isolation layers before mining, the water tank hot water was injected into the flexible isolation layer such that the iodized salt in the flexible isolation layer was completely dissolved, and the infrared monitor showed the yellow area in the image. At this point, the water flow monitoring Lines 1–3 and 5–7 show yellow status, indicating that after the fracturing of the isolation layer, the aquifer water flows downwards along the fracture. The lower part of monitoring Line 4 was compacted at the top of the coal seam, indicating that the cracks between the roof and the aquifer had not been communicated. Therefore, the water flow rate was 0 mL/min until the sixth periodic pressure. Mining was then undertaken on the working face. The No. 1 monitoring line was therefore less affected by mining due to its layout outside the stop line, and there was no significant change in water flow before the first grouting.

As shown in Fig. 13, when the working face progressed to second periodic pressure, with the collapse of the coal seam, the stress of the surrounding rock was redistributed, the height of the water flowing fractured zone of the roof increased, and the water flow of the No. 2 monitoring line increased from the initial 9.1 mL/min to 14.0 mL/min. As the working face was advanced above the No. 2 monitoring line, the fifth periodic of pressure were generated in the roof. The development height of the roof water flowing fractured zone reached 504.4 mm. The roof was separated and collapsed, the cracks in the monitoring line communicated with each other, and the rock stress was released. The water flow in the No. 2 monitoring line increased significantly. Monitoring line No. 3 was affected by advanced mining, resulting in the coal seam roof's increased rock fissures, the water flow path and resistance were reduced, and the water flow reached 48.3 mL/min. At the same time, the influence range of working face bedrock was close to the boundary of the first fracturing of the flexible isolation layer, and Groups 20–22 of isolation lays had been fractured.

When mining started at the sixth periodic pressure, the roof water-conducting fracture zone gradually reached the maximum height and penetrated the fractured isolation layer, and the fracture of the roof rock increased. Lines No. 2 and No. 3 reached 44.4 mL/min and 85.6 mL/min, respectively. In fact, the encounter may indicate that the confined water of the gritstone aquifer was released, and the water flow of the working face increased. Then, the working face progressed, and the collapsed gangue above the mining-out area was compacted into the bedrock roof. The stress in the goaf did not change significantly, and the cracks in the strata decreased. The No. 2 and No. 3 water flows of the monitoring line gradually dropped. During this period, the change law of monitoring Lines 4–7 was similar to that of No. 2 and No. 3. During coal seam mining, the roof underwent a process of fracture, collapse, compaction and full mining, and the water flow monitoring line also went through a process of rising and then falling.

When the working face was advanced to the eleventh periodic pressure, the grouting transformation of isolation layers 1–12 was conducted. The slurry was injected into the flexible isolation layer by hand pressure pump along the grouting pipe. After the slurry solidified, the colour of the No. 1 and No. 2 monitoring lines gradually became shallow, and the water flow gradually decreased under infrared observation. As the extraction of the coal seam progressed and the flexible insulation layer was broken and grouted, the colour of observation Lines 1–4 turned black in the infrared observation until the fourth grouting of insulation layer 18–19, and the water flow rate all showed 0 mL/min. However, the lower strata of the flexible isolation layer were not yet stabilized, so monitoring Lines 5–7 did not undergo any grouting transformation and still had a large water flow until the end of mining. Flow metre and infrared observations show that the destruction and grouting of the flexible isolation layer had a noticeable effect on the seepage characteristics of the overburden. In particular, after the grouting of the isolation layer, the slurry filled and solidified rapidly, the water flow decreased rapidly, and the water plugging effect of flexible isolation layer grouting was remarkable.

### Discussion and analysis

During coal seam mining, the fracturing of the flexible isolation layer should be based on the premining overtopping influence range; that is, when the boundary line of bedrock influence extends to the range of the flexible isolation layer reached by the fracturing area of the flexible isolation layer, the next fracturing should continue. The average boundary angle range of the bedrock was 76.7°, and the field angle should not be less than 73.57°. The grouting of the flexible isolation layer considers the full mining degree of the coal seam. When there is no significant change in stress in the mined area, grouting of the flexible isolation layer at the top of the goaf is conducted. According to the simulation experiment in this paper, the full mining distance of the working face is 1338.9 mm, and the actual distance on site is 187.446 m. It is calculated that the distance between the fracture of the flexible isolation layer should be no less than 854.8 mm away from the working face, and the actual distance on site is 119.672 m. After the working face enters full mining, the shortest distance between the fracturing grouting range of the flexible isolation layer and the working face is not less than 242.6 mm, and the actual distance on site is 33.964 m.

As seen from the previous analysis, with the advancement of the working face, the bedrock influence boundary angle of the coal seam does not change significantly, which only plays a guiding role in the fracturing sequence of the flexible isolation layer. The fracturing of the flexible isolation layer had an clear influence on the seepage of water-rich bedrock at the bottom of the Zhiluo Formation. The water-flowing fractured zone formed in the process of coal seam mining promoted the release of fractured water in the water-rich bedrock at the bottom of the Zhiluo Formation. The higher the height of the water-flowing fractured zone is, the greater the seepage of the water-rich bedrock. Coal seam mining had little effect on the seepage characteristics of the water-rich bedrock layer at the bottom of the Zhiluo Formation in the range of not disturbed by mining and advanced influence.

In accordance with the stress sensor data, when the working face passed a certain distance, the bottom plate of the extraction area was compacted by the falling gangue, and the sensor pressure data did not change with the mining face. At this time, the grouting of the fracturing area of the flexible isolation layer corresponding to the above goaf was not affected by the mining face. For example, the stress in the goaf of 1200 mm had no clear change. Therefore, the first grouting was conducted in the fracturing area. After the solidification of the grouting slurry, the water flow of monitoring lines No. 1 and No. 2 decreased significantly. This minimized the impact on the original geological environment and at the same time reduced the goaf water drainage of the working face. The sealing effect of the isolation layer has an important influence on promoting water-retaining coal mining.

The experimental application of the flexible isolation layer has realized its feasibility from the physical simulation test method in this paper. The realization of a flexible isolation layer requires premining fracturing and postmining isolation grouting. At present, premining fracturing can be achieved by directional drilling technology. There are also examples of roof separation grouting for postmining flexible isolation layer grouting^28,29^. Therefore, there is no technical bottleneck in field applications. Moreover, there is still a certain distance from the specific engineering application. According to the results of this study, it is predicted that the implementation of a flexible isolation layer will have great significance for water conservation coal mining in western China, which can reduce soil erosion and protect surface ecology.

## Conclusions

In this paper, an investigation on the mechanism of the multiphase and multifield coupling mechanism of deep mining in the western mining area was conducted. The whole process of the "flexible isolation layer" from fracturing to grouting transformation was realized through physical simulation, and the evolution law of the water-conducting fracture zone, stress variations, periodic roof pressure and overburden deformation characteristics were obtained for coal seam mining.

In terms of the challenges existing in conventional similar simulation materials, a new type of block similar material and flexible isolation layer similar material were innovatively developed, which have a simple structure, easy operation, cost and labour savings in the production process and can be widely used in similar simulation experiments of coal seam mining, laying an experimental foundation for the simulation of fracturing and grouting processes of flexible isolation layers in this paper.

In the experimental simulation, a total of four grouting transformations and six isolation layer fracturing cycles were conducted. The maximum height of the water-flowing fractured zone was 565.8 mm. The average bedrock influence boundary angle was 76.7°, and the average rock fracture angle in the goaf was 50.36°. The flexible isolated layer fracturing area should exceed the bedrock roof disturbance boundary, and the top collapsed rock layer in the mining void area must wait until its top collapsed rock layer is fully and adequately compacted before the isolated layer grouting transformation process can be carried out.

This physical simulation experiment successfully conducted the fracturing and grouting technology of a flexible isolation layer, verifying the feasibility of the technology of a flexible isolation layer forming a water isolation layer from fracturing to grouting reconstruction. Coal seam excavation had almost no effect on the seepage characteristics of overlying strata in the range of undisturbed mining and advanced influence. The increase in the water-flowing fractured zone and roof caving height caused by coal seam mining had an clear influence on the seepage characteristics of the roof, and the fracturing grouting of the flexible isolation layer had a significant influence on the seepage characteristics of the roof.

## References

[1] Sun W (2019). Development status and prospects of mine physical similar material simulation experiments. Geotech. Geol. Eng..

[2] Yin H (2016). Numerical simulation of water flow from the coal seam floor in a deep longwall mine in China. Mine Water Environ..

[3] Qiao W (2017). Effects of coal mining on shallow water resources in semiarid regions: A case study in the Shennan mining area, Shaanxi, China. Mine Water Environ.

[4] Zhao C (2019). Numerical simulation of the groundwater system for mining shallow buried coal seams in the ecologically fragile areas of Western China. Mine Water Environ..

[5] Liu S (2019). Fuzzy comprehensive risk evaluation of roof water inrush based on catastrophe theory in the Jurassic coalfield of northwest China. J. Intell. Fuzzy Syst..

[6] Liu S (2019). Zoning and management of phreatic water resource conservation impacted by underground coal mining: A case study in arid and semiarid areas. J. Clean. Prod..

[7] Wang J (2016). Effects of soil and topographic factors on vegetation restoration in opencast coal mine dumps located in a loess area. Sci. Rep..

[8] Xiaodan L (2019). Design strategy of water circulation system above and below ground in western mining area. Earth Environ. Sci..

[9] Sun Q (2017). Analysis and prevention of geo-environmental hazards with high-intensive coal mining: A case study in China's western eco-environment frangible area. Energies.

[10] Yao Q (2020). Discussion on coal and water co-mining in ecologically fragile mining areas in western China. Coal Sci. Technol..

[11] Sun Y (2020). Research progress of water environment, treatment and utilization in coal mining areas of China. J. China Coal Soc..

[12] Yang Z (2018). Classification of the type of eco-geological environment of a coal mine district: A case study of an ecologically fragile region in Western China. J. Clean. Prod..

[13] Chi M (2019). Simulation analysis of water resource damage feature and development degree of mining-induced fracture at ecologically fragile mining area. Environ. Earth Sci..

[14] Islam M (2009). ABM Finite element modeling of stress distributions and problems for multi-slice longwall mining in Bangladesh, with special reference to the *Barapukuria coal mine*. Int. J. Coal Geol..

[15] Newman C (2017). Assessment of potential impacts to surface and subsurface water bodies due to longwall mining. Int. J. Min. Sci. Technol..

[16] Scanlon B (2006). Global synthesis of groundwater recharge in semiarid and arid regions. Hydrol. Process.

[17] Gu D (2013). Water resource and surface ecology protection technology of modern coal mining in China's energy "Golden Triangle.". Strateg. Study CAE.

[18] Gu D (2015). Theory framework and technological system of coal mine underground reservoir. J. China Coal Soc..

[19] Wang S (2009). Study on coal mining for protecting ecological water level in the ecological fragile minging area. Metal Mine.

[20] Wang S (2010). Study on overburden aquclude and water protection mining regionazation in the ecological fragile mining area. J. China Coal Soc..

[21] Fan L (2015). Progress in engineering practice of water-preserved coal mining in western eco-environment frangible area. Coal Geol. Explor..

[22] Stachowski P (2018). Water reservoirs as an element of shaping water resources of post-mining areas. J. Ecol. Eng..

[23] Ma L (2019). Water conservation when mining multiple, thick, closely-spaced coal seams: A case study of mining under Weishan Lake. Mine Water Environ..

[24] Meng G (2020). Physical simulation experiment on prevention and control of water inrush disaster by backfilling mining under aquifer. Environ. Earth Sci..

[25] Wang P (2018). Physical simulation of mining effect caused by a fault tectonic. Arab. J. Geosci..

[26] Xu S (2020). Impacts of aquitard properties on an overlying unconsolidated aquifer in a mining area of the loess plateau: Case study of the Changcun colliery, Shanxi. Mine Water Environ..

[27] Zhang S (2018). Physical simulation research on evolution laws of clay aquifuge stability during slice mining. Environ. Earth Sci..

[28] Feng YJ (2012). Test on hard and stable roof control by means of directional hydraulic fracturing in coal mine. Chin. J. Rock Mech. Eng..

[29] Yang J (2021). Application of long borehole directional drilling hydraulic fracturing technology in buertai coal mine of Shendong coal group. Coal Geol. Explor..

